# Is There a Role of Cupping Therapy in the Treatment of Carpal Tunnel Syndrome in Primary Care Setting?

**DOI:** 10.7759/cureus.12954

**Published:** 2021-01-28

**Authors:** Ayaaz Farhat, Saqib M Mughal

**Affiliations:** 1 Genetics, Alpha Medical Practice, Birmingham, GBR; 2 Family Medicine, Alpha Medical Practice, Birmingham, GBR

**Keywords:** wet cupping, dry cupping, carpal tunnel syndrome, hijama, neuropathy

## Abstract

Carpal tunnel syndrome (CTS) is a clinical syndrome, which causes significant morbidity. It is currently managed conservatively with splinting or steroidal injections. Where conservative management is unsatisfactory, surgical decompression is carried out. Because of dangerous complications of surgical procedures and increasing economical burden of CTS condition, safe, traditional, cost effective conservative adjunct option is being evaluated in this review article. Cupping therapy is an ancient but increasingly popular therapy for a variety of pathologies. Studies over the last decade have indicated potential therapeutic value of cupping therapy for the management of CTS. Whilst there is some biomedical rationale supporting the usage of cupping therapy in CTS the evidence is not sufficient to support the inclusion of cupping therapy in management of carpal tunnel syndrome in formal pathways. High quality trials with increased participant numbers, development of blinded study options and a regulatory body for cupping therapy are required so that cupping therapy can be established as a potent therapeutic option for CTS. The review was conducted to examine the efficacy of cupping therapy in management of CTS.

## Introduction and background

Carpal tunnel syndrome

Carpal tunnel syndrome (CTS) is a compression neuropathy commonly diagnosed in the upper limbs. Symptoms of CTS are not linear and hence patient may present with a variety of symptoms [1,2]. There are various criteria used for diagnosis and prevalence being dependent on it, 3% to 6% of the general population is known to be suffering from it [1,2]. According to Pays de la Loire study, prevalence decreased between 2004-2011 from 3.35 to 2.98 per 1000 person-years over a period of eight years for surgically treated CTS and from 5 to 3 per 1000 person-years for work-related diseases CTS category [3]. Middle-aged individuals are mostly affected by CTS showing peak incidence at 55-60 years. Usually, females are more prone to CTS as compared to males [4]. CTS symptom severity reported by patients is significantly and associated with anxiety, depression, quality of life, and societal costs despite adjustment for smoking, drinking, comorbidities, age, gender and body mass index [5].

People who are working in industries suffer from occupational hazards, especially when they repetitively apply high force or pressure with their hands, they are more prone to develop CTS. Use of vibrating tools at industry also contributes in the development of CTS. Incidences reported by medical literature for general population and workers who use their hand and wrist frequently are 1% and 5%, respectively [6]. There are two distinct varieties of CTS: acute and chronic. The acute form is uncommon as it is associated with rapid and sustained rise of pressure in carpel tunnel. Additionally, acute form of CTS is associated with fracture radius [7].

Various non-occupational causes of CTS can be classified into local, regional, and systemic. Local causes include inflammatory, tumours, trauma, and anatomical defects. Regional causes include osteoarthritis, amyloidosis, gout and rheumatoid arthritis. Systemic causes are obesity, menopause, hypothyroidism, diabetes, pregnancy, systemic lupus erythematosus, renal failure, scleroderma, acromegaly, dermatomyositis, long-term haemodialysis, leukemia, multiple myeloma, sarcoidosis, alcoholism, and haemophilia [6].

There is limited data on the economic impact of CTS. However, an American study in 2007 suggested the income loss per CTS patient was estimated at $45,000-89,000 over a period of six years when it was compared with controls [8].

Cupping therapy is an ancient method of applying quick, vigorous, rhythmic force for improving blood supply. It is used for various neuropathy conditions. Hence, there is need of reviewing cupping therapy as an alternative treatment option for CTS as it is cost effective and traditional healing option.

## Review

Pathophysiology

Nerve conduction impairment can be explained by many theories, but mechanical compression, micro-vascular insufficiency and theory of vibration are the most popular ones. Mechanical compression theory states that compression of the median nerve in the carpal tunnel gives rise to the symptoms of CTS. Underlying aetiology of mechanical compression is not considered in this theory which is a major drawback [9-12]. The compression of nerve is mediated by several factors like strain by exertion, overuse of wrist joint, hyperfunction, repeated or prolonged wrist extension, prolonged grasping and unaccustomed manual work [9]. Another popular theory of micro-vascular insufficiency explains about the lack of blood supply to the nerve because of which the normal function of transport of nutrients and oxygen gets affected that can lead to loss of nerve’s ability to transmit nerve impulses [10,11].

Clinical features

Clinically, CTS can present with a variety of neuropathic symptoms due to the median nerve compression. The sensory component of median nerve is involved in early stage and symptoms include intermittent pain and paraesthesia in the hand corresponding to the distribution of the median nerve. Classically, the thumb, index and middle fingers and radial half of the ring finger are involved. Other features such as wasting of the thenar eminence musculature, reduced grip strength aid in making a clinical diagnosis of the syndrome [13,14].

Signs

Several tests help in the diagnosis of CTS. While none of these tests are diagnostic on their own but they are complementary to each other in making an accurate diagnosis. The various tests are Tinel’s sign, Phalen’s sign, square wrist sign, pressure provocation test, closed fist sign, flick sign, flexion and extension of wrist test, Katz hand diagram, and tourniquet test.

Diagnosis

The diagnosis of carpal tunnel syndrome (CTS) is done based on its characteristic symptoms and electrodiagnostic studies confirm it. Reliability of diagnosis can be increased by using sonography. It is mainly based on an increase in cross-sectional area of the median nerve at the level of the pisiform or hamate bone. However, electrodiagnostic study in practice is gold standard for diagnosis. We can’t replace electrodiagnostic studies with sonography, but sonography may serve as an additional investigation [15].

Current treatment and limitations

In recent practice, treatment is selected as conservative or surgical. This is based on stage of the disease, the severity of the symptoms and the patient's preference. Oral corticosteroid treatment, local corticosteroids injection into the carpal tunnel, and splinting are proven effective treatment options in early stages in some individuals [16].

Surgical treatment is considered when conservative treatment fails. Open carpal tunnel release (OCTR) is surgery of choice and it has high success rate for any type of CTS. For minimal invasion, nowadays people consider endoscopic carpal tunnel release as it can give same results as OCTR with less scar, less pain, better recovery. Surgical procedures have their own complications such as transection of the flexor tendons, superficial palmar arch or even the ulnar nerve, median nerve, mostly because of technical errors [17].

Though the prognosis of non-surgical treatment options is served by poor medical literature, up to 70% of cure rate can be achieved with steroids alone and hence steroid therapy is given prior to surgery to increase the effectiveness of severe cases. Overall prognosis of the treatment options is not satisfactory if we look from the community perspective. Published data available on patient-reported success rates post-surgery can be used to summarize CTS prognosis as seen in Table 1 below [18].

**Table 1:** Overall prognosis of carpal tunnel syndrome (CTS) cases with different treatment options

Treatment	Complete cure	Slight improvement	Worse
Surgery	75%	17%	8%
Local corticosteroid injection	70% (relapse within 1 year)	30%	<0.1%
Splinting	30%	70%	0
No treatment	20%	40%	40%

Hence, our focus should be on to study other conservative traditional and complementary methods for generation of medical evidences around it so that one can decide whether to use them solely or in combination with the standard treatment options currently available [18].

Cupping therapy

**Figure 1:**
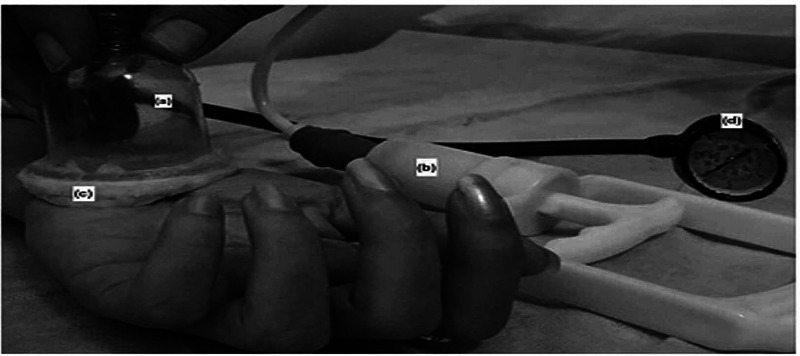
Cupping instrument. (a) Cup, (b) Silicone interface, (c) Suction pump, (d) Pressure gauge

Cupping is an ancient method for the treatment of aches and pains associated with various diseases. It consists of application of quick, vigorous, and rhythmical strokes on the skin with the help of suction cup which stimulate cutaneous, subcutaneous muscles and increases blood flow in that area. Hence, it has potential to enhance the quality of life with minimal side effects. Cupping is described as dry or wet cupping. In the dry cupping skin is pulled into the cup via vacuum pressure whereas in the wet cupping, scarification is also carried out in order to draw blood into the cup (Figure 1) [19].

Several theories have been tried to explain the mechanisms by which cupping therapy provides clinical benefit in a wide range of pathologies [20]. Changes in biomechanical properties of the skin reduce the pain and it is explained by the “Pain-Gate Theory” (PGT), “Diffuse Noxious Inhibitory Controls” (DNICs), and “Reflex Zone Theory” (RZT). “Nitric Oxide Theory” explains muscle relaxation, specific changes in local tissue structures and increase in blood circulation. “Activation of Immune System Theory” (AIST) explains the immunomodulatory effects of cupping therapy. “Blood detoxification theory” explains release of toxins and removal of wastes and heavy metals [19].

Of these described theories, in the context of CTS, the most relevant to consider is the nitric oxide (NO) theory. This theory postulates that via NO-mediated endothelial vasodilation, cupping essentially can improve circulation in a given anatomical area. Cupping therapy releases NO from endothelial cells and, induces beneficial biological changes. This mechanism of action is explained by “Release of nitric oxide and increased blood circulation theory”. It was reported in a trial that increased expression of nitric oxide synthase(s) was higher around skin acupuncture points of rats [21]. Notably, endothelium-derived relaxing factor (EDRF), the active substance from the perfusate during application of the stimulus, has been identified as NO. Vasculatures release EDRF which mediates the vasodilation of endothelial linings. Further, the action of NO on vascular smooth muscle is similar to EDRF [22]. Nitric oxide synthesis is critical aspect for wound collagen accumulation and mechanical strength. Numerous studies proved that cupping dilates topical capillaries and increases dermal blood flow [23,24]. Vasodilators like adenosine, noradrenaline and histamine contribute to vasodilation at the area of cupping which leads to increased blood circulation [25]. Because of cupping therapy, endothelial cells secrete NO which gives certain effect necessary for prevention of atherosclerosis [19].

It is the improved circulation in the carpal tunnel which would thus reduce the level of compression on the median nerve and its blood supply resulting in clinical improvement.

Review of current evidence

Carpal tunnel syndrome is a condition in which interstitial pressure inside the canal increases leading to lower capillary blood flow. We found limited evidence-based literature on cupping therapy for CTS. Literature search was conducted on PubMed for only English language articles in indexed journals. Two randomized control trials (RCTs) by Michalsen et al. and Mohammadi et al. on cupping therapy for CTS and two case reports are available in medical literature so far [26-29].

Among RCTs, effect of wet cupping therapy on CTS was demonstrated by Michalsen et al. in his RCT conducted on 52 patients in 2009 [26]. In 26 patients of study population, the trapezius muscle area for wet cupping therapy, which consists of scarification (puncturing) of the skin, application of vacuum cup and mechanical suction, was chosen. Twenty-two controls were treated by application of heating pad. The change in total CTS symptom severity from day 0 to 7 was the primary outcome of the study. The visual analog scale (VAS) was used to assess the symptom score (pain, tingling and numbness). Secondary outcomes considered the functional impairment which was assessed by standardised DASH questionnaire. Symptom severity score before therapy was 61.5 ± 20.5 which dropped significantly to 24.6 ± 22.7 mm at day 7 in the intervention group. For control group, change in symptom severity score was from 67.1 ± 20.2 to 51.7 ± 23.9 mm. Repeated measurement ANCOVA gave group difference as 24.5 mm (95% CI -36.1; -2.9, P < .001). The secondary outcomes of functional severity demonstrated statistically significant improvements in the group receiving cupping treatment vs the control group. Patients with CTS who were treated with wet cupping experienced reduction in pain and other symptoms. Treatment group patients also noted improved functional ability, quality of life and reduced associated neck pain for at least one week, but since sample size of this study is limited, this evidence is not strong enough to apply this therapy in clinical practice. Further head-on trials with current standard treatment options are needed to establish the power of medical evidence for specifically ‘wet’ type of cupping therapy for CTS [26].

Per se RCT for dry cupping for CTS was done by Mohammadi et al. In this study, routine physiotherapy consisted of transcutaneous electrical nerve stimulation and ultrasound which was given to both the intervention and control groups of 28 patients each [27]. In intervention group, apart from routine physiotherapy, cupping was applied on the wrist for 4 min without any scarification. Levine’s CTS questionnaire was used to assess symptom severity and functional status of patients. Distal latency for motor and sensory components was also measured with nerve conduction studies. The treatments were carried out on alternate days for a total of 10 sessions. Results were analysed after 20 days showing a statistically significant improvement in symptom severity for decrease in distal sensory latency in the test group (routine physiotherapy with cupping) as compared with the control group. Unlike Michalsen study, result of this study was not consistent with the functional severity aspect, probably because of the difference in the type and site of the cupping as well as the patient selection. Mohammadi et al. study gave implication for physiotherapy practice of cupping therapy to a routine therapy as it improves the distal sensory disturbance of the median nerve.

Per se case study on suction decompression published in 2019 was of a 27-year-old CTS patient [28]. Right hand was showing CTS symptoms of progressive numbness and tingling in the thumb, index finger and middle finger over 6-8 month. Cupping therapy was self-administered in this case on her affected wrist at home over three months. She was advised to carry out cupping on her wrist for 3-5 minutes daily. Within 1st week, she noted improvement in symptoms and complete resolution of the symptoms was noticed after 6-8 weeks. At three months when she was reviewed, there was improvement in distal latency, ultrasound appearance as well as clinical improvement in symptoms. Hence, suction decompression being self-administered provides a viable alternative treatment option for patients with mild CTS.

Another case of wet cupping therapy by Aboonq reported a work-related CTS case of 37-year-old Egyptian male in 2019 [29]. This report is the only report discussing wet cupping therapy over the volar wrist. The patient was given medical therapy of analgesic anti-inflammatory drugs and muscle relaxants initially but the results were not satisfactory. Instead of option surgery, the patient opted wet cupping (Al-hijamah) as an alternative option. This case was of severe type diagnosed based on electromyography (EMG) and nerve conduction velocity studies (NCV). Post treatment, there was improvement in the EMG and NCV studies, and the degree of CTS was improved from severe to moderate. Clinically the patient’s symptoms improved significantly and clenched fist formation was possible and pain and numbness in distal fingers had resolved. Hence this Al-hijamah can be used as an adjuvant to pharmacological treatment modalities.

Two RCTs by Huisstede et al. and Al Bedah et al. are given for the evidence for non-surgical treatments for CTS and systematic review on wet cupping therapy, respectively [20,30]. Al Bedah et al. mentioned that cupping therapy can be used for CTS but highlighted the limitations in blinding for study design.

The authors and respective study results for wet cupping therapy have been summarised in Table 2.

**Table 2:** Summary of authors and respective study results for cupping therapy in the treatment of carpal tunnel syndrome.

Author, Year	Study type	N (Control + Test)	Treatment		Results
Michalsen et al., 2009 [26]	Randomized clinical trial study	52 (26 + 26) participants	Control group	Test group	On follow-up after seven days, the test group reported: 1. Significant reduction in severity of symptoms. 2. Significant reduction in neck pain. 3. Significant reduction in disability. Improvement in quality of life was reported only by the test group.
			Single local application of heat	Single application of wet cupping	
Mohammadi et al., 2019 [27]	Randomized clinical trial study	56 (28+28) hands	Control group	Test group	Compared to the control group, test participants reported significant: 1. Improvement in severity of symptoms. 2. Decrease in distal sensory latency.
			Routine physiotherapy	Routine physiotherapy and cupping therapy	
Sucher, 2019 [28]	Case study	1 participant (1 hand)	Daily use of cupping device		Follow-up after three weeks: 1. Initial wrist abnormalities were resolved. 2. Patient was asymptomatic during the 3-month follow-up visit.
Aboonq, 2019 [29]	Case study	1 participant (2 hands)	Arabic wet cupping therapy (Al-hijamah)		1. Immediately after treatment: Patient was able to clench his fist and felt no pain, numbness or paresthesia. 2. Follow-up after few days: Patient was almost symptom free. 3. Follow-up after three years: Patient was comfortable with tolerable mild pain.

Discussion

The current study aimed to assess whether we can think about cupping therapy for the management of CTS. Studies have found strong association of CTS with comorbidities, age, gender, BMI, psychological distress, but work-related CTS of factory worker is major unavoidable concern [5].

Among the treatment options available for CTS, moderate satisfactory outcomes of surgery are reported but chances of dreadful complications can’t be void. Initially, physician chooses conservative approach for management but since there are very limited options available, new approaches need to be considered [17].

Cupping has been part of the art of healing methods for over 5000 years in countries like China, the Middle East, Ethiopia or Central and North Europe. As the advances in modern cell pathology occurred, cupping was slipped out of the physician’s choice of treatment and so is true with other humoral therapeutic techniques. In Germany, it was revived attention on parts of these methods. Nowadays, German lay healers are practicing these methods. In the US, acupuncture specialists treat low back pain in patients with cupping therapy [31].

As already discussed, scientific rationale to explain why cupping therapy would show benefit in the management of CTS is because of the NO-mediated vasodilation. It improves the circulation in the area of carpal tunnel; hence, we can easily achieve reversal of the compression defects like CTS. Increasing number of systematic reviews of cupping therapy on pain conditions like low back pain, hypertension, herpes zoster, facial paralysis are reported [32-35].

## Conclusions

There is growing evidences on cupping therapy in general in the management of pain conditions and carpal tunnel syndrome being the entrapment condition can be resolved by cupping. There is a paucity of well controlled RCTs for cupping therapy as a treatment option for CTS, but for other diseases this treatment option is well documented. The review was conducted to examine the efficacy of cupping therapy in management of CTS.

In the primary care setting where resources are limited for surgical intervention, the usage of cupping therapy as add-on therapy along with medical management can be an effective method of treatment. There is also a need of regulatory body for cupping therapy. A standardized procedure with established protocols will be beneficial as well as defining contraindications and complications for cupping therapy. We should request regulatory authorities and government to form a taskforce to consult with and set standard guidelines should be there for medical professionals and regulation of practitioners so that quality is assured which further may lead to evidence-based practice.
